# Microglial state transitions are constrained by developmental and metabolic checkpoints

**DOI:** 10.3389/fncel.2026.1852176

**Published:** 2026-05-15

**Authors:** Adil El Mesaoudi, Dong Won Kim

**Affiliations:** 1 Danish Research Institute of Translational Neuroscience (DANDRITE), Nordic EMBL Partnership for Molecular Medicine, Aarhus University, Aarhus, Denmark; 2 Department of Biomedicine, Aarhus University, Aarhus, Denmark

**Keywords:** checkpoint, developmental licensing, metabolic capacity, microglia, neurodegeneration, neuroimmunology, state transitions, tissue gating

## Abstract

Single-cell transcriptomic and epigenomic profiling has revealed extensive microglial heterogeneity across development, homeostasis, and disease. However, one recurring observation remains unexplained. Only subsets of microglia adopt expected transcriptional programs in a given context, and transcriptional activation often fails to translate into functional execution. Current frameworks describe microglial states, but they do not explain why individual cells differ in their ability to access, sustain, or complete state transitions. Here, we propose a checkpoint framework for microglial state transitions. In this model, transitions depend on prerequisite conditions that must be met before a response can proceed. We use the term checkpoint in the sense of cell-cycle biology, where progression depends on prior conditions, rather than in the signal-integration sense used for peripheral immune checkpoints. We first consider the peripheral checkpoint model, in which transitions are driven mainly by activating and inhibitory receptor signaling. In that system, continuous hematopoietic renewal buffers many individual cell-level constraints. We then argue that this logic is modified in microglia. Their embryonic origin, lifelong residence in the CNS, and limited replacement capacity make cellular history a more durable determinant of responsiveness. Within this framework, we define three non-redundant classes of checkpoint-like constraints. The first is developmental licensing, which establishes accessible response space through lineage specification, postnatal maturation, and age-dependent stabilization. The second is metabolic and proteostatic capacity, which determines whether cells can meet the energetic and protein-handling demands required for execution. The third is tissue-derived gating, in which neuronal, astrocytic, and extracellular matrix signals set permissive or non-permissive conditions for transition. Together, these constraints help explain why microglial responses are heterogeneous, why transcriptional state and functional output can diverge, and why disease-associated profiles may reflect stalled or incomplete transitions rather than stable functional identities. This framework shifts attention from descriptive states to constrained transitions. It also suggests that therapy should focus on restoring transition competence rather than simply suppressing or inducing specific microglial states.

## 1 Introduction: from descriptive states to constrained transitions

Single-cell transcriptomic and epigenomic studies have transformed our understanding of microglial biology. They reveal extensive heterogeneity across development, homeostasis, and disease (Li et al., 2019; Li X. et al., 2024; Masuda et al., 2019; Young et al., 2021; Martins-Ferreira et al., 2025). These approaches defined states linked with synaptic remodeling, lipid handling, and neurodegeneration (Keren-Shaul et al., 2017; Ulland et al., 2017; Kim et al., 2022; Zia et al., 2022; De Schepper et al., 2023; Prakash et al., 2025). Ongoing work continues to subdivide microglial populations with greater resolution (Tuddenham et al., 2024; Fumagalli et al., 2025; Snijders et al., 2025). Yet a fundamental question remains. Why do only subsets of microglia engage specific responses in a given context? A transcriptional state does not explain how it is accessed. It does not explain whether it can be sustained. It does not explain why only a fraction of cells adopt it under shared conditions. Consistent with this gap, microglial responses are often uneven and incomplete (Tuddenham et al., 2024; Martins-Ferreira et al., 2025). In disease, only a subset of microglia adopts characteristic transcriptional programs (Keren-Shaul et al., 2017; Ulland et al., 2017; Kim et al., 2022). Others remain in homeostatic or intermediate/maladaptive states despite similar exposure to pathology (Fumagalli et al., 2025; Snijders et al., 2025). Moreover, transcriptional activation does not reliably translate into functional execution, even within responding populations (Ulland et al., 2017; Ayata et al., 2018; Gazestani et al., 2023; Sun et al., 2023). Cells may upregulate expected gene programs but fail to complete phagocytosis, sustain metabolic remodeling, or resolve inflammatory signaling. This disconnect between signal exposure and response completion suggests that key regulatory constraints operate upstream of, or independently from, extracellular cues (De Schepper et al., 2023; Liu et al., 2023; Yin et al., 2023; Li X. et al., 2024; Saeki et al., 2024). The central question should therefore be what determines whether a given cell has the competence to execute the program that the transcriptional state implies.

We propose that this constrained responsiveness can be understood through a checkpoint framework. In this model, specific prerequisites must be met before a state transition can proceed. These include developmental, metabolic, and niche-derived factors. We use “checkpoint” in the sense defined in cell-cycle biology. It refers to a barrier where progression depends on prior conditions, not just signal input (Hartwell and Weinert, 1989; Morgan, 2007). Applied to microglia, this means that an activating stimulus is not sufficient. A cell may receive the signal but fail to transition if the required conditions are not met. This usage differs from the peripheral immune definition of a checkpoint. There, the checkpoint function is framed as a balance between inhibitory and activating signals. We develop this distinction in the following sections.

This framework must be modified from peripheral immune checkpoint logic, which we describe in Section 2, to account for the unique biology of microglia. Their embryonic origin, lifelong CNS residence, and limited capacity for replacement shift the logic of responsiveness. In this setting, individual cellular history becomes a dominant determinant, and population dynamics play a more limited role. In the following sections, we adapt peripheral checkpoint logic to this context. We outline three classes of prerequisite constraints: developmental licensing, metabolic and proteostatic capacity, and tissue-derived gating. Together, these define the range of accessible microglial state transitions. This reframing shifts the focus from discrete states to constrained transitions and provides a mechanistic basis for selective responsiveness in health and disease. It also highlights more principled targets for therapeutic intervention.

## 2 Peripheral immune checkpoint logic and its assumptions

The peripheral immune checkpoint model is best characterized in T cells. Here, receptor-ligand interactions determine whether cells initiate proliferation (Smith-Garvin et al., 2009; Marchingo et al., 2014), acquire effector functions (Joshi et al., 2007; Pipkin et al., 2010; Kaech and Cui, 2012), or enter states such as exhaustion (Im et al., 2016; Sen et al., 2016). Within this framework, transitions are threshold-dependent. Sufficient activating input, combined with relief of inhibitory signaling, drives progression into a defined functional state (Altan-Bonnet and Germain, 2005; Feinerman et al., 2008; Pardoll, 2012; Sharpe and Pauken, 2018). Signal integration is the primary regulatory variable, and the system is tuned to detect input strength while maintaining robustness to noise.

A key structural feature is continuous population renewal. Peripheral immune cells are generated from hematopoietic progenitors (Orkin and Zon, 2008; Seita and Weissman, 2010). They undergo cycles of expansion and contraction in response to antigen exposure (Kaech et al., 2002; Seder et al., 2008). Therefore, checkpoint regulation shapes both individual decisions and population composition over time. Signal integration is then coupled to selection (Jenkins et al., 2001; Blattman et al., 2002), in which the design provides resilience. When exhausted or dysfunctional cells accumulate, the system retains a route to recovery. Newly generated progenitor-derived cells with intact responsiveness can be recruited. In some contexts, pre-exhausted progenitor populations sustain responses even when terminally exhausted effectors cannot be reinvigorated (Im et al., 2016; Siddiqui et al., 2019). Checkpoint-targeted therapies exploit this feature, and inhibitory signaling works in part because a reservoir of less-exhausted or newly generated cells exists to respond (Barber et al., 2006; Im et al., 2016).

This does not mean that peripheral immune transitions are unconstrained at the level of individual cells. Classical studies established that brief signaling can be sufficient to commit cells to effector programs (van Stipdonk et al., 2001; Curtsinger et al., 2003), suggesting broad cell-intrinsic competence. However, subsequent work has shown that metabolic state can strongly gate effector differentiation and function (Pearce et al., 2009; Chang et al., 2013). Epigenetic imprinting can stabilize dysfunctional or lineage-committed states, particularly in chronic stimulation settings such as exhaustion (Im et al., 2016; Sen et al., 2016). Tissue residency can also impose durable constraints, as seen in tissue-resident memory programs that couple retention, adaptation, and function to local environmental signals (Skon et al., 2013; Mueller et al., 2014; Iijima and Iwasaki, 2015; Amsen et al., 2018). Thus, peripheral immunity also contains metabolic, epigenetic, and tissue-dependent forms of gating. The important point is not that such constraints are absent, but that they are embedded within a system that can compensate for them through turnover, recruitment, and population replacement.

Accordingly, the peripheral checkpoint framework rests less on the assumption that every individual cell is fully competent than on the assumption that functional competence remains sufficiently available at the population level. Intracellular capacity is often limiting for particular cells, and tissue-derived signals can strongly shape accessible programs. However, because the peripheral immune system continually regenerates and redistributes cells, these constraints do not usually define a fixed ceiling on overall responsiveness. Instead, they are buffered by population dynamics, allowing similar functional programs to be deployed across changing contexts and tissues (Masopust et al., 2001; Gebhardt et al., 2009; Schenkel et al., 2014; Beura et al., 2016; Park et al., 2019).

## 3 Why microglia diverge from peripheral immune checkpoint logic

As outlined in Section 2, the peripheral checkpoint model accommodates individual cell-level constraints, including metabolic gating, epigenetic imprinting, and tissue-derived regulation. However, these constraints are buffered by continuous population renewal, such that functional competence remains available at the system level (Fujiwara et al., 2021; Moreno Ayala et al., 2023). Microglia do not follow this logic. Their responsiveness is constrained by pre-existing properties that shape how signals are interpreted in the first place. These properties are established during development, shaped by cellular history, and continuously modulated by the tissue environment (Mathys et al., 2017; Gazestani et al., 2023; Sun et al., 2023; Yin et al., 2023; Feiten et al., 2026). In this respect, microglia share features with other tissue-resident macrophages, a comparison we develop below.

As a result, checkpoint-like mechanisms in microglia do not regulate selection among competent cells. They determine whether individual cells are competent to respond at all. This distinction has direct implications for heterogeneity, dysfunction, and therapeutic targeting.

The most fundamental divergence concerns ontogeny and turnover. Peripheral immune cells are continuously replenished from hematopoietic progenitors (Rossi et al., 2005; Orkin and Zon, 2008). Dysfunctional or exhausted cells can be supplemented or replaced by newly generated cells with intact functional capacity. Relieving inhibitory signaling can restore responsiveness at the population level, even when individual cells are compromised. Microglia are long-lived, self-renewing, and developmentally specified (Utz et al., 2020; Barry-Carroll et al., 2023; Yu et al., 2023). Opportunities for replacement by monocyte-derived cells are limited, and their functional repertoire is established early and maintained over time. Relieving inhibitory signaling at the receptor level may therefore be insufficient. It cannot restore access to states that were not established or that have become inaccessible through aging or prior activation (Utz et al., 2020; Carver et al., 2023; Fixsen et al., 2023). What appears reversible in peripheral systems may reflect deeper, less reversible constraints in microglia.

The divergence from peripheral immune biology becomes clearer when microglia are compared with other tissue-resident macrophage populations (Lazarov et al., 2023). Kupffer cells, alveolar macrophages, and Langerhans cells are also long-lived and partly self-renewing populations with embryonic contributions (Hashimoto et al., 2013; Yona et al., 2013; Ginhoux and Guilliams, 2016). However, many peripheral tissue-resident macrophage pools receive monocyte-derived input during inflammation, aging, or tissue remodeling, and the balance between embryonic and monocyte-derived cells varies across tissues and disease states (Bain et al., 2014; Scott et al., 2016). Microglia sit at the more constrained end of this spectrum. The blood–brain barrier, limited monocyte-derived replacement under homeostatic conditions, and long-term stability of the adult microglial pool leave fewer population-level routes for restoring competence. Thus, other tissue-resident macrophages may share elements of checkpoint-like regulation, but microglia represent a particularly stringent case because replenishment provides less buffering.

Returning to the divergences from peripheral checkpoint logic, a second concerns the link between signal reception and functional execution. Signal-execution uncoupling is not unique to microglia. In peripheral systems, chronically stimulated T cells can upregulate activation markers while remaining functionally impaired (Im et al., 2016; Sen et al., 2016). Similarly, chronically stimulated monocytes and monocyte-derived macrophages can retain activation markers while showing impaired functional output, as seen in endotoxin tolerance and monocyte dysfunction during sepsis or chronic inflammation (Biswas and Lopez-Collazo, 2009; Seeley and Ghosh, 2017). However, this uncoupling typically arises from sustained signaling history and can be bypassed at the population level through recruitment of less-exhausted or newly generated cells. In microglia, signal reception and execution are often uncoupled (Marino et al., 2023; Feiten et al., 2026). Cells may receive appropriate stimuli yet fail to complete transitions, and limitations in metabolic, proteostatic, or transcriptional capacity can block execution (Mathys et al., 2017; Gazestani et al., 2023; Sun et al., 2023). This results in partial or stalled responses that lack corresponding functional output. Checkpoint regulation in microglia cannot be understood solely at the level of receptor signaling. It must account for whether the cell has the internal capacity to implement what the signal instructs.

An additional divergence lies in tissue context. Tissue-derived signals shape peripheral immune responses and can impose durable functional constraints, as demonstrated by tissue-resident memory programs (Skon et al., 2013; Mueller et al., 2014; Amsen et al., 2018). However, these signals are typically not the primary determinant of whether a response can proceed. They modulate the character of responses within a system where cell-intrinsic competence and population-level renewal remain the dominant regulatory variables. In the central nervous system (CNS), these signals can become major determinants of competence because microglia are permanently embedded within a structured niche. Inputs from neurons, astrocytes, and extracellular matrix define what responses are possible (Stogsdill et al., 2022; Wang H. et al., 2022; Mallach et al., 2024; Ferrari-Souza et al., 2026). These signals are spatially and temporally structured. Identical receptor-level inputs can therefore produce different outcomes depending on context (Yin et al., 2023; Feiten et al., 2026). This distributes checkpoint regulation across the tissue rather than localizing it to individual cells.

Temporal dynamics further distinguish microglia from peripheral immune cells. Path dependence is not absent from peripheral immunity. Epigenetic imprinting during chronic stimulation can stabilize dysfunctional states in individual cells (Im et al., 2016; Sen et al., 2016). However, peripheral responses are typically structured around episodic activation and reset, and population renewal limits the cumulative impact of individual cell histories on system-level output (Fujiwara et al., 2021; Moreno Ayala et al., 2023). Microglial responses unfold over longer timescales and are shaped by cumulative cellular history, including development (Utz et al., 2020; Fixsen et al., 2023), aging (Li X. et al., 2024; Li B. et al., 2024), and prior stress or injury (Perry and Holmes, 2014; Ellwanger et al., 2021; Kim et al., 2024). This introduces path dependence, meaning the response to a stimulus depends on the prior state. Identical inputs can produce divergent responses across a microglial population depending on its history, violating the assumption established in peripheral checkpoint models that competence is broadly available once signaling thresholds are met (Wendeln et al., 2018; Hata et al., 2023).

Finally, in peripheral systems, dysfunction at the individual cell level does not necessarily constrain the system. Even when terminally exhausted cells cannot be reinvigorated, progenitor-derived or less-exhausted populations can sustain or restore responses (Im et al., 2016; Siddiqui et al., 2019). The system-level consequence of individual dysfunction is therefore limited. In microglia, dysfunction more often reflects underlying structural constraints. These include incomplete developmental licensing (Utz et al., 2020; Han et al., 2023), limited metabolic or proteostatic capacity (Ulland et al., 2017; Nugent et al., 2020; Wu et al., 2021), and persistent tissue-derived inhibition (Yin et al., 2023; Bedolla et al., 2024). These are not inhibitory overlays that can simply be removed, but define boundaries on accessible states that can be reached from a given cellular starting point. Attempts to directly translate peripheral checkpoint blockade strategies to microglia, therefore, risk misreading constrained competence as reversible suppression (Kimura et al., 2025). Such approaches may relieve signaling constraints without restoring the capacity required to respond.

**Figure 1:**
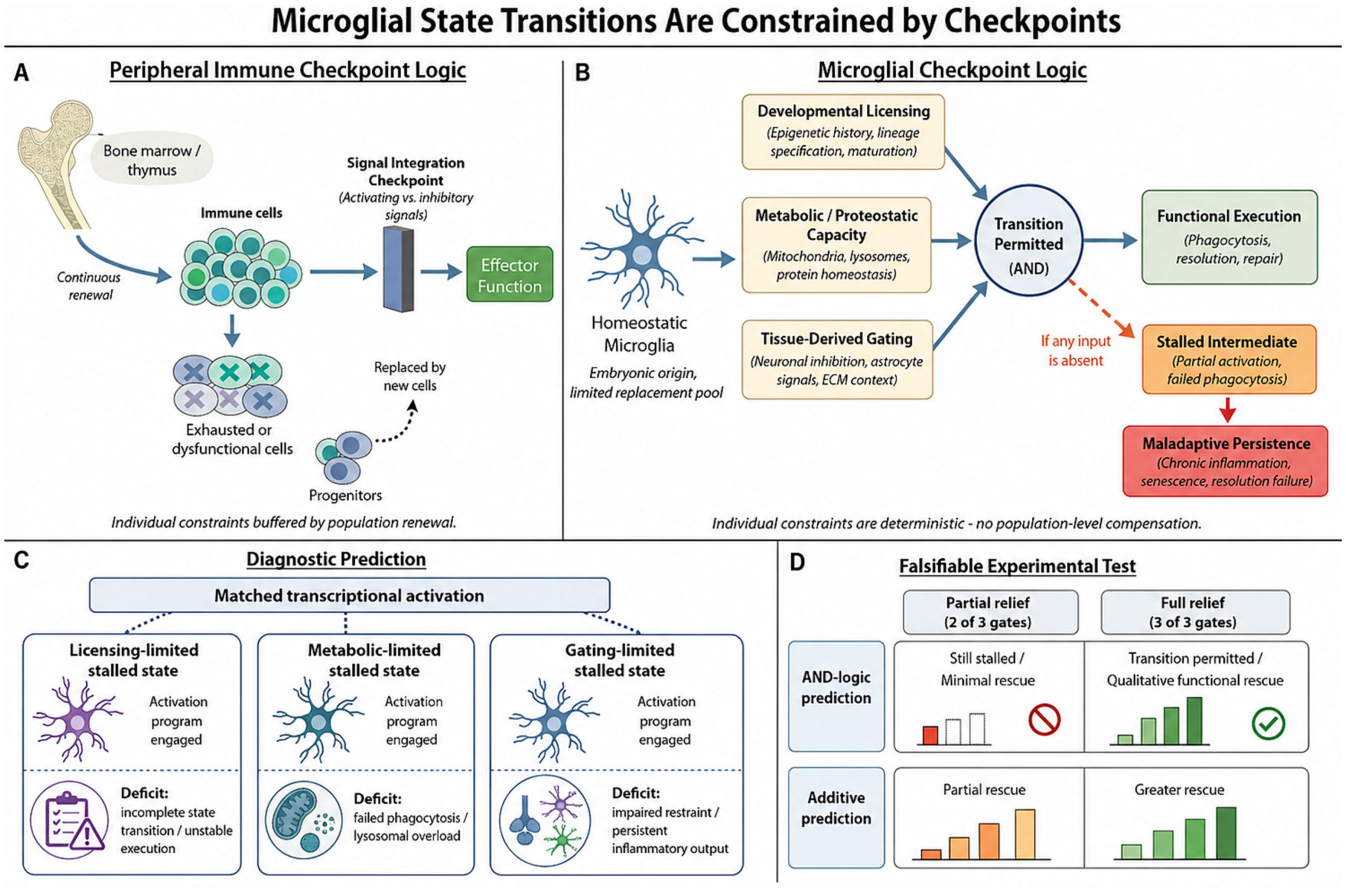
Checkpoint-constrained model of microglial state transitions. **(A)** In peripheral immune systems, state transitions are primarily regulated by signal integration at receptor-level checkpoints. Cell-intrinsic constraints such as metabolic capacity, epigenetic history, and tissue-derived conditioning are buffered at the population level by continuous renewal from hematopoietic progenitors, allowing exhausted or dysfunctional cells to be replaced. **(B)** In contrast, microglia originate from embryonic precursors and are maintained as a largely self-renewing population with limited replacement. Productive microglial state transitions, therefore, require the simultaneous satisfaction of three prerequisite constraints: developmental licensing, metabolic/proteostatic capacity, and tissue-derived gating. These inputs converge on an AND-like transition-permission node. When all three constraints are satisfied, microglia can execute functional responses such as phagocytosis, resolution, and repair. If any prerequisite is limiting, cells may enter stalled intermediate states that can progress to maladaptive persistence. **(C)** The model predicts that distinct stalled states can exhibit matched transcriptional activation while differing in the limiting constraint and associated functional deficit. Licensing-limited, metabolic-limited, and gating-limited stalled states may therefore appear similar by activation markers alone but differ in transition stability, phagocytic/lysosomal competence, or inflammatory restraint. **(D)** The model is experimentally falsifiable. Under AND-like logic, relief of two constraints is predicted to produce limited or unstable rescue if the third remains unmet, whereas satisfaction of all three prerequisites should permit a qualitative functional transition. In contrast, an additive model predicts graded improvement as individual constraints are relieved.

Together, these differences reframe microglial checkpoint regulation as a problem of cellular competence rather than signal integration (Figure 1). The question is therefore not only which signals microglia receive, but whether the developmental, metabolic, and tissue conditions required to execute those signals are in place.

### 3.1 Checkpoint logic versus existing frameworks

The framework advanced here overlaps with, but is not reducible to, existing concepts of priming, epigenetic memory, and metabolic gating. Each of these describes how microglial responsiveness can be biased. Priming describes how prior exposure shifts the magnitude or kinetics of subsequent responses (Perry and Holmes, 2014; Wendeln et al., 2018). Epigenetic memory describes how chromatin modifications acquired during development or chronic stimulation stabilize biased transcriptional programs (Gosselin et al., 2017; Hamagami et al., 2025). Metabolic gating describes how bioenergetic and biosynthetic limits constrain which activation states can be sustained (Ulland et al., 2017; Marschallinger et al., 2020). Each is empirically well supported and accounts for a specific class of observation.

The checkpoint framework makes a distinct claim. Microglial state transitions require the simultaneous satisfaction of three prerequisite classes that are analytically separable, even though they interact biologically: developmental licensing, metabolic and proteostatic capacity, and tissue-derived gating. We refer to this as an AND-logic architecture, because transition completion is predicted to require all three prerequisite classes to be sufficiently satisfied. These classes are therefore proposed to function as non-redundant prerequisites rather than merely additive contributors or hierarchically ordered modulators. Failure at any class is predicted to produce a stalled intermediate rather than a proportionally reduced response. This generates a testable prediction that neither priming, epigenetic memory, nor metabolic gating makes on its own. Transcriptional activation and functional execution should decouple systematically at points of checkpoint failure, and the pattern of decoupling across axes should indicate which prerequisite is unmet. Under additive or hierarchical models, partial relief of constraints should produce graded improvement. Under the checkpoint model, relief of one or two constraints should produce limited or unstable rescue if another prerequisite remains unmet. This distinguishing prediction defines the framework empirically, not merely conceptually. Within-class coupling, such as the interdependence of metabolic and proteostatic systems described in Section 4.2, is compatible with the claim that the three prerequisite classes are functionally distinguishable at the level of transition failure.

A central term in the framework developed here is functional competence, which we use in a specific and operational sense. Competence refers to the capacity of a cell to execute a defined functional program in response to an appropriate stimulus, and is operationalized as the correspondence between markers of transcriptional activation and matched functional outputs under defined conditions. Functional outputs include sustained phagocytic throughput measured over biologically relevant timescales, resolution kinetics of inflammatory signaling, metabolic flux sufficient to support the response, such as oxygen consumption rate or lipid oxidation capacity during lipid handling, and proteomic indicators of completed biosynthetic programs rather than initiated ones. Under this definition, a cell that displays transcriptional activation but fails to produce the corresponding functional output is transcriptionally activated but not functionally competent. This dissociation is the empirical signature that the checkpoint framework predicts at points of constraint failure. Competence is therefore not a fixed intrinsic trait, but a context-dependent condition that holds when the relevant prerequisite classes are satisfied. This definition makes competence measurable in principle and falsifiable as a predictor of functional outcome; Section 6 discusses the experimental platforms through which it can be assessed in practice.

A related clarification concerns the relationship between cellular competence and signal integration more broadly. Competence is not independent of signaling in the causal sense; signals received during development and homeostasis construct the regulatory architecture that defines competence. The framework claims that on the timescale of an acute response, this architecture is fixed relative to the acute signal, such that acute signaling operates within rather than reconfigures the space of accessible states. Cellular competence and acute signal integration are therefore distinct regulatory variables, not because they are causally independent, but because they operate on separable timescales that become relevant at different points in a response.

## 4 Checkpoint-like constraints

The three constraint classes described below: developmental licensing, metabolic and proteostatic capacity, and tissue-derived gating, implement the AND-logic architecture introduced in Section 3.1. A successful state transition is predicted to require sufficient satisfaction of all three; failure at any single class is predicted to produce a stalled intermediate. Each subsection below defines one prerequisite class and specifies how its failure may stall transition completion.

### 4.1 Developmental licensing checkpoints

Microglial state transitions are not freely accessible. They are constrained by developmental programs that define which responses a cell can execute (Utz et al., 2020; Fixsen et al., 2023). Unlike peripheral immune cells, microglia originate from embryonic yolk sac-derived precursors (Utz et al., 2020; Barry-Carroll et al., 2023). They undergo a defined sequence of maturation within the CNS (Masuda et al., 2022; Han et al., 2023). These processes do more than generate transcriptional diversity. They establish stable regulatory states that persist into adulthood, defining which functional programs remain accessible. Therefore, developmental licensing can be viewed as a checkpoint, meaning it is a developmental establishment of accessible response space. Early ontogenetic events are converted into long-term constraints on responsiveness. Critically, constraint does not imply rigidity, but it means that plasticity remains and operates within defined boundaries. Accessible transitions are shaped by prior history, not uniformly open. Within the AND-logic architecture defined above, developmental licensing functions as the first prerequisite: without the relevant developmental history, a cell may remain unable to complete the corresponding response, even when metabolic capacity and tissue context are otherwise favorable.

An initial layer of constraint is imposed during lineage specification and early CNS entry (Utz et al., 2020; Brioschi et al., 2023). Primitive macrophages that seed the brain acquire an identity distinct from circulating monocytes (Shay et al., 2013; Fixsen et al., 2023), including the establishment of core regulatory circuits that are maintained over time. This early specification limits access to alternative macrophage programs available to peripheral cells (Li et al., 2019; Van Hove et al., 2019). At the same time, it preserves adaptive capacity within a microglia-specific regulatory space. A second constraint emerges during postnatal maturation (Masuda et al., 2022), when microglia undergo regionally patterned differentiation in response to local signals. These include neuronal activity, myelination, and circuit remodeling (Favuzzi et al., 2021; Stogsdill et al., 2022; McNamara et al., 2023). These programs stabilize distinct regulatory configurations (Van Hove et al., 2019). The epigenetic modifications acquired during this period persist into adulthood (Saeki et al., 2024; Hamagami et al., 2025). As a result, adult transitions occur within pre-existing limits and do not reflect complete reprogramming (Leyns et al., 2017; Lan et al., 2024). Plasticity within these limits remains possible and has been demonstrated previously (Gosselin et al., 2017; Saeki et al., 2024; Hamagami et al., 2025). However, it is conditional on the existing regulatory landscape.

A further layer of constraint is introduced through the progressive restriction of proliferative and differentiation potential. Early in development, microglia retain considerable flexibility (Li et al., 2019; Barry-Carroll et al., 2023). They can expand and adopt diverse phenotypes in response to environmental signals. With maturation and aging, this flexibility narrows. Homeostatic programs stabilize, and the epigenetic landscape becomes more fixed (Li B. et al., 2024; Li X. et al., 2024; Hamagami et al., 2025). This limits large-scale reconfiguration in response to injury or disease (Hu et al., 2021; Carver et al., 2023). This progressive narrowing is itself graded rather than absolute. Some degree of state-specific plasticity is retained in the adult brain, but it operates within an increasingly restricted range. Microglia must adapt within a largely fixed population, which limits but does not eliminate the possibility of reversing dysfunctional states under appropriate conditions.

The molecular basis of developmental licensing lies in the coordinated establishment of microglia-specific regulatory architecture. Lineage-defining transcription factors, including PU.1/SPI1 and SALL1, establish and maintain the microglial regulatory landscape during CNS colonization and postnatal maturation (Kierdorf et al., 2013; Buttgereit et al., 2016; Masuda et al., 2022). SALL1 in particular functions as a microglial identity factor whose loss permits drift toward a macrophage-like state, illustrating how specific transcription factors enforce the developmental boundary between microglia and peripheral lineages (Buttgereit et al., 2016). In combination with signal-dependent factors such as MAFB and CEBP family members, these transcriptional regulators help establish enhancer repertoires that shape accessible response programs (Gosselin et al., 2017; Holtman et al., 2017). These enhancer landscapes are further stabilized by chromatin-regulatory mechanisms, including polycomb-associated repression at developmentally restricted loci and BAF-associated control of chromatin accessibility at microglia-relevant enhancers (Saeki et al., 2024; Hamagami et al., 2025; Veremeyko et al., 2019; Gong et al., 2022). Once established, these configurations can persist through cell division and constrain the transcriptional programs available to adult microglia (Gosselin et al., 2017; Lan et al., 2024). Path dependence therefore operates, at least in part, through stable enhancer and chromatin configurations acquired during licensing, which define a bounded space of transcriptionally accessible states. This provides a molecular substrate for the licensing constraint: acute stimulation acts within a pre-existing regulatory landscape rather than freely opening all possible response trajectories.

Developmental licensing also determines the accessibility of reactive states observed in disease (Perry and Holmes, 2014; Wendeln et al., 2018). Microglial states associated with neurodegeneration or demyelination are not uniformly induced across all cells (Keren-Shaul et al., 2017; Mathys et al., 2017). Instead, their accessibility depends on prior maturation and context-specific priming (Ellwanger et al., 2021; Lan et al., 2024). This contingency is consistent with the constraint framework. These programs are accessible only to cells with the appropriate regulatory history, not simply because they are induced by stronger activation. Cells lacking this prior licensing may fail to respond despite an equivalent signal (Van Hove et al., 2019; Silvin et al., 2022). By contrast, cells that have been appropriately primed may transition more readily, illustrating that constraint and facilitation are two sides of the same developmental process.

Together, these observations support a constrained model of microglial heterogeneity. States reflect developmental trajectories rather than unrestricted interconversion. Early specification, postnatal maturation, and age-dependent stabilization define a hierarchy of permissible responses. This hierarchy persists across the lifespan, meaning plasticity remains, but it is conditional and bounded. The therapeutic implication is not that developmental constraints are fixed. It is that interventions aimed at modulating microglial states must account for the regulatory history that determines which transitions are within reach. Understanding how these developmental checkpoints are established and modified will be essential for redirecting microglial function in disease.

### 4.2 Metabolic and proteostatic capacity

As the second prerequisite class in the AND-logic architecture, metabolic and proteostatic capacity is distinct from developmental licensing in mechanism and timescale but equivalent in status. A developmentally licensed cell exposed to permissive tissue-derived signals is still predicted to fail at transition completion if its metabolic or proteostatic capacity is insufficient. The tight coupling between metabolic and proteostatic systems discussed later in this section occurs within this prerequisite class and is compatible with the broader claim that the three classes are functionally distinguishable at the level of transition failure. Microglial state transitions are constrained by metabolic and proteostatic capacity. Cells must meet the energetic and protein-handling demands of a given response (Ulland et al., 2017; Nugent et al., 2020; Sung et al., 2025), and transcriptional changes alone are not sufficient to define a functional state (Gazestani et al., 2023; Sun et al., 2023). Transitions require coordinated increases in metabolic flux, organelle activity, and protein turnover to support execution (Wu et al., 2021; Di Fraia et al., 2024). When these systems are limited, transitions fail despite appropriate external cues. As with developmental licensing, this does not mean that metabolic and proteostatic constraints are fixed or uniformly invincible. Rather, they define thresholds that vary across cells and conditions, and that determine whether a given cell can act on the signals it receives. Metabolic and proteostatic capacity may therefore function as intrinsic checkpoints. They do not control signal reception, but rather determine whether those signals can be translated into functional outcomes.

A primary constraint arises from the coupling between metabolic configuration and function. Homeostatic microglia operate within a relatively stable metabolic regime that supports surveillance and baseline maintenance (Lee J. et al., 2023; Fumagalli et al., 2025). Reactive states impose substantially higher energetic and biosynthetic demands. Phagocytosis requires local protein synthesis at the site of engulfment to sustain membrane remodeling and cargo processing (Vasek et al., 2023; Jacquet et al., 2024). Cytokine production demands rapid biosynthetic output that strains ribosomal and secretory capacity (Roy et al., 2020; Wang C. et al., 2022). Lipid handling requires mitochondrial reorientation toward oxidative substrates to support the energetic cost of lysosomal degradation (Marschallinger et al., 2020; Lee S. et al., 2023). Meeting these demands requires coordinated shifts in substrate utilization (Marino et al., 2023), mitochondrial activity (Ryu et al., 2024; Yamauchi et al., 2024), and redox balance (Flury et al., 2025). These shifts are not uniformly accessible. Microglia may in some contexts exhibit more limited metabolic flexibility compared with peripheral myeloid cells (Yin et al., 2023; Raju et al., 2025), reflecting their long-term adaptation to the CNS and a self-renewing population structure. As a result, only a subset of cells can sustain the metabolic reconfiguration required for full activation. Others remain in partial or non-productive states, not because they lack the instructive signal, but because they cannot meet the energetic cost of execution.

Proteostatic capacity imposes a parallel constraint. Reactive programs require rapid synthesis of secreted factors (Roy et al., 2020; Wang C. et al., 2022), membrane proteins (McQuade et al., 2025), and lysosomal components (Wu et al., 2021; McQuade et al., 2025). They also require increased protein turnover during phagocytosis and stress responses. A concrete example is the lysosomal recycling burden imposed by amyloid engulfment. Microglia attempting to clear fibrillar amyloid must sustain high lysosomal throughput over extended periods. When degradative capacity is insufficient, undigested material accumulates intralysosomally, triggering stress responses that divert resources away from the activation program (Choi et al., 2023). These demands place coordinated load on the endoplasmic reticulum, unfolded protein response, autophagy-lysosomal system, and proteasome (Choi et al., 2023; Duran-Aniotz et al., 2023; Di Fraia et al., 2024). If these systems are insufficient, cells accumulate misfolded or damaged proteins, activate stress pathways, and stabilize in dysfunctional intermediate states. Proteostasis may act as a prerequisite for transition rather than a downstream consequence of activation. Restoring proteostatic capacity can re-enable stalled transitions by enhancing autophagic flux, providing one example (Choi et al., 2023).

Molecular nodes governing metabolic and proteostatic capacity are identifiable and provide discrete points of intervention. On the metabolic side, the TREM2-DAP12-PI3K-mTOR axis couples receptor engagement to biosynthetic capacity and mitochondrial function, and loss of TREM2 signaling is associated with reduced ATP availability and impaired sustained responses (Ulland et al., 2017; Wang et al., 2015). Mitochondrial regulators, including PGC-1α and TFAM, shape biogenesis and oxidative capacity (Nugent et al., 2020), while lipid-handling pathways involving LPL, ABCA1, and LXR-PPARγ influence whether microglia can process myelin- or amyloid-associated lipid loads without entering dysfunctional lipid-laden states (Marschallinger et al., 2020; Loving and Bruce, 2020). On the proteostatic side, integrated stress response components such as PERK and GCN2, autophagy regulators such as TFEB, and lysosomal regulators including TFE3 represent candidate nodes whose activity can influence whether cells sustain the biosynthetic and degradative loads of reactive programs (Choi et al., 2023; Di Fraia et al., 2024; Duran-Aniotz et al., 2023; Iyer et al., 2022; Lu et al., 2019; Zhao et al., 2025). Together, these examples support the framework’s claim that metabolic and proteostatic capacity can act at the level of execution, shaping whether transcriptional activation is translated into sustained functional output.

Metabolic and proteostatic constraints are tightly coupled. Mitochondria supply ATP and regulate redox state and biosynthetic intermediates (Ryu et al., 2024; Yamauchi et al., 2024). Lysosomal activity supports both degradative capacity and recycling of substrates required for metabolic processes (Wu et al., 2021; Choi et al., 2023). A clear illustration of this coupling is lipid overload. When microglia accumulate excess lipid, lysosomal pathways become saturated. This impairs recycling of fatty acid substrates needed to sustain mitochondrial oxidative metabolism, which in turn reduces the ATP available for continued phagocytic activity (Marschallinger et al., 2020; Prakash et al., 2025; Sung et al., 2025). The result is a coupled bottleneck in which neither system can recover independently, and cells become trapped in intermediate states. Importantly, this bottleneck is graded and conditionally reversible, but not absolute. Interventions targeting either lysosomal capacity or mitochondrial function have been shown to at least partially restore transition competence (Ulland et al., 2017; Choi et al., 2023).

These limitations become more pronounced with aging and in chronic disease. Accumulation of mitochondrial damage, reduced autophagic flux, and sustained activation of stress responses progressively lower the threshold below which microglia can execute demanding functional programs (Carver et al., 2023; Li B. et al., 2024; Li X. et al., 2024). A well-characterized consequence is mitochondrial stress-induced senescence. Sustained energetic insufficiency drives cells into a state characterized by reduced phagocytic output and elevated inflammatory signaling that is resistant to resolution (Yamauchi et al., 2024; Hu et al., 2025). Under these conditions, cells often initiate but do not complete state transitions, resulting in partial activation that lacks corresponding functional output. This provides a mechanistic explanation for why similar stimuli produce divergent responses depending on cellular condition. The limiting factor is the ability to sustain the required metabolic and proteostatic changes, not signal availability. Peripheral immune systems can compensate for such limitations through rapid cell turnover or recruitment of new cells. Microglia must meet these demands within a relatively fixed population in a constrained environment. This potentially enforces a tighter link between internal capacity and state accessibility. However, restoring capacity can reopen blocked transitions.

Viewed this way, at least some microglial states observed in disease may reflect failed or incomplete execution of functional programs rather than stable, intrinsically distinct identities. Cells that cannot meet the metabolic or proteostatic demands become trapped in intermediate states that contribute to pathology. Therapeutic strategies should therefore target cellular capacity. Restoring mitochondrial function, lysosomal activity, or protein homeostasis may enable completion of appropriate transitions. The goal shifts from altering state identity to restoring competence.

### 4.3 Tissue-derived gating

Microglial state transitions are further constrained by continuous signals from the CNS microenvironment. To define this constraint, it is necessary to distinguish tissue-derived gating from general homeostatic regulation. All cells are subject to ongoing environmental influence, and not every contextual signal functions as a gate. We reserve “gating” for tissue-derived permissive or non-permissive signals that determine whether a transition can proceed at all. These signals are necessary but not sufficient for transition. Their removal or modification permits change, but does not drive it. By contrast, contextual signals modulate rate or magnitude, but do not define accessibility. A gate sets a prerequisite condition, and a context variable adjusts a parameter. Tissue-derived signals in the CNS function as gates in this specific sense. They establish permissive or non-permissive conditions before and independently of any activating stimulus. Their effects can be inhibitory, permissive, or instructive depending on the signal class and cellular context. Unlike peripheral immune cells that traffic between tissues, microglia are permanently embedded within a structured niche in the CNS (Tremblay et al., 2011; Michelucci et al., 2018). Because this embedding is permanent, gating is likely to be constitutive rather than episodic. It continuously defines what the cell can do, regardless of incoming signals. As the third prerequisite class, tissue-derived gating is analytically separable from developmental licensing and metabolic/proteostatic capacity, even though these processes interact biologically. A developmentally competent cell with adequate internal capacity is still predicted to fail at transition completion if tissue-derived gating conditions are non-permissive. Conversely, favorable tissue signals may be insufficient to restore transition completion when developmental licensing or execution capacity is limiting. This is the specific relationship among the three classes proposed by the AND-logic architecture.

One well-studied gate is neuronal inhibitory signaling (Lyons et al., 2007; Zhang et al., 2011; Lehrman et al., 2018). Interactions mediated by neuronal surface molecules and soluble factors actively suppress inflammatory and phagocytic programs under homeostatic conditions (Wang et al., 2015; Lehrman et al., 2018; Hu et al., 2025). This establishes a tonic inhibitory baseline, which is mechanistically distinct from simple signal competition and functions as an inhibitory gate. Transition into reactive states requires the reduction or withdrawal of this inhibition (Zhang et al., 2011; Liu et al., 2019). The process is graded rather than binary (Liu et al., 2019; Preszler et al., 2026). The magnitude and spatial distribution of neuronal inhibition define the local threshold. These thresholds determine whether microglia can initiate or sustain a response, such that transitions remain restricted while inhibitory tone exceeds a critical level.

Astrocytes provide an additional regulatory layer, though the nature and hierarchy of astrocyte-derived signals are less resolved than that of neuronal inhibition. Astrocyte-derived cytokines and growth factors can function in a permissive capacity. They establish conditions that engage disease-associated programs. In some contexts, astrocyte-dependent priming is required for full microglial engagement with pathological stimuli (Liddelow et al., 2017; Vainchtein et al., 2018; Nguyen et al., 2020). Other astrocyte-derived signals appear instructive, actively directing the character of microglial responses rather than enabling them (Zhu et al., 2026). Astrocytic regulation of extracellular ion concentrations and neurotransmitter availability can further modulate microglial responsiveness. This alters the biochemical environment in which signals are interpreted. Together, these observations support a role for astrocytes in regulating microglial competence. However, it should be noted that the evidence remains more fragmentary. A clear functional hierarchy among astrocyte-derived signals has not been established. These signals should therefore be treated as likely gates rather than fully defined ones.

Oligodendrocytes and myelin also contribute to tissue-derived gating of microglial responses, although the signals involved are less well defined than those from neurons or astrocytes. Myelin acts both as cargo that microglia must handle and as a source of permissive or inhibitory cues. For example, exposed phosphatidylserine can promote myelin debris recognition and clearance, whereas sialylated myelin-associated ligands can engage CD33 and related Siglec receptors to restrain microglial activation or phagocytosis (Claude et al., 2013; Griciuc et al., 2013). Oligodendrocyte-derived factors can also influence microglial state during myelination and remyelination, with reciprocal signaling between the two populations regulating myelin debris clearance and the timing of repair responses (Cantuti-Castelvetri et al., 2018; Lloyd et al., 2019). In demyelinating disease, altered oligodendrocyte-microglia signaling may both lift inhibitory constraints and introduce cues that bias microglia toward states that fail to resolve, consistent with the gating logic proposed here. These mechanisms remain incompletely resolved, and oligodendrocyte-derived gating should therefore be treated as a likely but less-characterized component of the tissue-derived constraint class.

Spatial variation across the CNS introduces a further dimension to gating. Differences in neuronal composition, myelination, vascular structure, and extracellular matrix generate region-specific environments that differentially constrain microglial responses (Grabert et al., 2016; De Biase et al., 2017). Regional response differences likely reflect a combination of local gating conditions and the intrinsic developmental and activation history of resident microglia. These two sources of variation are not fully separable from current data and should not be treated as mutually exclusive. However, the key contribution of tissue-derived gating is that it imposes constraints that cannot be reduced to cellular history alone. Two microglia with equivalent developmental licensing and metabolic capacity may nonetheless differ in transition accessibility if they reside in regions with distinct neuronal inhibitory tone, astrocyte-derived signaling, or extracellular matrix composition. Gating thus defines a layer of regulation that operates orthogonally to cell-intrinsic competence, even when the two are difficult to disentangle empirically.

In disease, tissue-derived constraints are altered but not removed. Injury or neurodegeneration can reduce neuronal inhibitory tone and lower activation thresholds (Lehrman et al., 2018; Preszler et al., 2026; Zhang et al., 2011; Wang et al., 2015). Changes in astrocyte function (Liddelow et al., 2017; Guttenplan et al., 2020) or extracellular composition (Bedolla et al., 2024; Ferrari-Souza et al., 2026) can further permit or restrict specific responses. These changes are often spatially heterogeneous and temporally incomplete. As a result, microglia may satisfy gating conditions only partially, initiating transitions but failing to complete them. Disease-associated states can thus be understood as reflecting altered gating conditions rather than simply unrestricted activation. This distinction has therapeutic relevance, since restoring appropriate gating may be as important as targeting cell-intrinsic activation pathways. This mode of regulation differs fundamentally from peripheral checkpoint models, where tissue context is largely modulatory. In the CNS, gating is distributed across the tissue and arises from coordinated interactions among multiple cell types (Grabert et al., 2016; Liddelow et al., 2017; Vainchtein et al., 2018), making state accessibility inherently context-dependent and resistant to single-target intervention.

Viewing tissue-derived signals as gating mechanisms reframes microglial heterogeneity. Cells operate within a spatially organized and cell-type-distributed set of prerequisite conditions. Effective modulation of microglial behavior in disease must act at two levels. Cell-intrinsic pathways must be targeted, and the tissue environment must also be engaged. At the same time, the boundaries of tissue-derived gating remain incompletely defined, which remains an active area of investigation.

## 5 Implications for disease and therapy

If microglial states reflect constrained transitions rather than freely accessible endpoints, then many disease-associated states may be better understood as failures or maladaptive transitions. Cells may fail to enter, complete, or resolve appropriate response programs. This framing shifts emphasis away from transcriptional signatures and toward the mechanisms that determine whether transitions can occur. On this view, disease-associated microglial states would instead reflect blocked, incomplete, or maladaptive transitions, and are not stable identities to suppress. Distinguishing these possibilities in specific disease contexts remains an open empirical challenge.

One implication is that similar transcriptional signatures observed across diseases may not indicate a shared program (Krasemann et al., 2017; Zhou et al., 2020; Yang et al., 2021), but could instead reflect common constraints on state transitions. Existing frameworks, particularly the disease-associated microglia/homeostatic dichotomy, treat these signatures as evidence of a shared activation program with a defined functional identity (Keren-Shaul et al., 2017; Krasemann et al., 2017). The constraint model makes a different prediction: convergent transcriptional profiles may arise not from a common program, but from common bottlenecks. Cells stalled at similar gating barriers will resemble each other transcriptionally without sharing an underlying functional trajectory. Furthermore, diseases, injury, and even natural aging can produce overlapping microglial profiles (Mathys et al., 2017; Kenigsbuch et al., 2022; Zia et al., 2022; Wachter et al., 2024). However, the underlying causes may differ (Gerrits et al., 2021; Ennerfelt et al., 2022; Coburn et al., 2025). In one context, microglia may initiate a reparative program but fail to complete it due to metabolic limits (Ulland et al., 2017; Marschallinger et al., 2020). In another, persistent inhibitory signals from the tissue environment may prevent effective engagement of phagocytic functions (Yin et al., 2023; Bedolla et al., 2024). Further, developmental or age-related restrictions may limit the available response repertoire (Utz et al., 2020; Carver et al., 2023; Fixsen et al., 2023). The same apparent state could arise from distinct blocked transitions, complicating attempts to assign uniform functional or therapeutic meaning to recurring signatures. Biomarkers of functional competence and transition capacity may therefore be more informative than state identity alone. Such measures may better support disease stratification and intervention design.

This perspective also helps clarify why microglial responses can be both protective and pathogenic (d’Errico et al., 2021; Ennerfelt et al., 2022; Odfalk et al., 2022). Transitions that are beneficial at early stages of injury may become detrimental if sustained beyond their appropriate duration (Mathys et al., 2017; Roy et al., 2020; Wang C. et al., 2022). Conversely, failure to initiate these transitions can impair clearance or repair (Ulland et al., 2017; Huang et al., 2021; Choi et al., 2023). The critical variable is not the identity of a given state. It is whether transitions are properly regulated in time and magnitude. The checkpoint framework suggests that disease arises when this regulation is misaligned. Cells either fail to enter required programs or fail to terminate them appropriately. Though the causal direction of this misalignment in human disease remains to be established in most contexts.

These considerations suggest a reorientation of therapeutic strategy around three conceptual therapeutic classes, with each targeting a distinct class of constraint. The first is restoration of metabolic and proteostatic capacity. Broad suppression of microglial activation is unlikely to be effective if pathology reflects failed execution but not excessive activation. Inhibiting inflammatory pathways may reduce harmful outputs in the short term. However, it can also impair debris clearance, trophic support, or circuit remodeling if these depend on transient activation (Pluvinage et al., 2019; Spangenberg et al., 2019; Kiani Shabestari et al., 2022). Interventions that enhance lysosomal throughput, restore mitochondrial function, or stabilize proteostasis take a different approach. They instead aim to enable completion of transitions that have stalled (Ulland et al., 2017; Choi et al., 2023). The second is the modification of tissue-derived gating. Where inappropriate inhibitory signals prevent microglia from engaging necessary responses, selectively reducing that inhibition may restore access to specific transitions without triggering non-specific inflammation. Examples include CD22 blockade, which restores phagocytic function in aged microglia by relieving an age-associated inhibitory signal (Pluvinage et al., 2019), and CD33 modulation, which alters microglial engagement with amyloid (Griciuc et al., 2013). Conversely, when gating has been pathologically loosened, for example through reduced CD200-CD200R inhibitory tone in aging or neurodegeneration, restoring appropriate inhibitory input may be as important as targeting cell-intrinsic activation pathways (Walker et al., 2009; Wang et al., 2020). This latter direction is less developed experimentally and should be viewed as a conceptual therapeutic class rather than a validated strategy. The third is restoration of resolution capacity. Many pathological microglial states reflect failure to terminate responses rather than excessive activation. Specific resolution mechanisms have been identified that, when engaged, can redirect sustained inflammatory signaling toward termination. These include anti-inflammatory lipid mediators such as specialized pro-resolving mediators, which promote phagocytic clearance while dampening cytokine production (Serhan and Levy, 2018; Julliard et al., 2022), and signaling through receptors such as TREM2 and TAM family receptors that couple engulfment activity to the downregulation of inflammatory gene programs (Huang et al., 2021; Ennerfelt et al., 2022). Autophagy-dependent clearance of inflammatory signaling complexes represents a further resolution mechanism whose impairment has been linked to chronic microglial activation (Choi et al., 2023). In these cases, restoring termination mechanisms may be more effective than blocking activation (Mathys et al., 2017; Roy et al., 2020). Where the first archetype addresses failure to complete a transition, this third archetype addresses failure to exit one. A cell that lacks the capacity to resolve an inflammatory program requires a different intervention than one that cannot initiate phagocytosis.

A further implication concerns the limits of competence restoration, meaning not all constraints are reversible. If developmental licensing was never established, restoring metabolic or signaling capacity may not be sufficient. Similar limits may arise with aging or chronic stress. In such cases, replacement of endogenous microglia with competent progenitor-derived or induced pluripotent stem cell (iPSC)-derived cells may bypass intrinsic constraints (Mancuso et al., 2019; Xu et al., 2020). This approach would address constraints that are not accessible to pharmacological modulation of existing cells. However, it introduces its own challenges around engraftment, regional specification, and the re-establishment of appropriate tissue-derived gating relationships. Cell replacement is not a near-term therapeutic option in most contexts, but the developmental constraint framework predicts its relevance. When the limiting factor resides within the cell rather than the environment, replacement may be required.

This model also suggests that therapeutic efficacy will depend on disease stage. Early in disease, when constraint architecture may remain modifiable, interventions may still redirect microglial responses toward repair (Pluvinage et al., 2019). At later stages, prolonged stress, aging, or tissue remodeling may impose more rigid constraints that limit microglial adaptability (Carver et al., 2023; Sun et al., 2023; Li X. et al., 2024). This may explain the gaps between preclinical success and clinical outcomes. Interventions effective in acute or early-stage models fail in chronic human disease, indicating that timing is likely to be integral to therapeutic strategy. However, the rate at which constraint architecture becomes resistant to modification in human disease is not yet well characterized. In addition, an important alternative is that some disease-associated microglial states are not stalled intermediates but stabilized terminal adaptations. Distinguishing these models requires longitudinal and functional measurements rather than transcriptomics alone.

Finally, these considerations caution against direct translation of peripheral immune checkpoint strategies to microglia. Releasing inhibitory signaling may not restore function if the primary limitation lies in an inaccessible developmental state space or insufficient execution capacity. In some cases, such interventions may exacerbate dysfunction by pushing cells into unsustainable or incomplete responses (Kimura et al., 2025). Effective therapy must integrate multiple levels of control. Receptor signaling must be considered alongside developmental constraints, intracellular capacity, and tissue context. The goal is not to impose predefined microglial states, but to restore the conditions that allow appropriate responses to occur and resolve.

### 5.1 Scope, alternative explanations, and limits of the framework

The framework advanced here is proposed as a general architecture for microglial state regulation, but its explanatory scope and distinguishability from alternative accounts require explicit bounding.

The checkpoint framework is most directly motivated by chronic neurodegenerative contexts in which microglial responses are sustained, heterogeneous, and apparently uncoupled from functional outcome: Alzheimer’s disease, related tauopathies, progressive multiple sclerosis, and age-associated dysfunction (Keren-Shaul et al., 2017; Mathys et al., 2017; Gerrits et al., 2021; Ennerfelt et al., 2022). In these contexts, long disease duration, incomplete resolution, and discordance between transcriptional activation and effective clearance are the observations the framework is built to explain. The framework may also apply to other chronic CNS conditions with comparable features, including chronic neuroinflammation and repeated injury. Its applicability is less clear in acute contexts such as stroke, acute traumatic injury, or acute viral encephalitis, where response kinetics are compressed and population-level dynamics may differ. The framework should also not be directly extrapolated to peripheral macrophage or monocyte biology without independent evaluation, because continuous renewal and distinct ontogenetic histories alter the constraint landscape (Section 3).

Several alternative explanations for incomplete microglial responses exist and are not excluded by the checkpoint framework. Stochastic variability in single-cell responses, arising from fluctuations in transcription, receptor-ligand kinetics, or cell-cycle state, predicts variable outcomes without persistent structure across cell populations (Elowitz et al., 2002; Raj and van Oudenaarden, 2008). Microenvironmental gradients in oxygen, substrate availability, or signaling molecule concentration predict graded responses tracking spatial position. The checkpoint framework is distinguishable from both: under stochastic variability alone, transcription–function dissociation should be random and unstructured; under gradient-based explanations, dissociation should track continuous spatial variables. The framework instead predicts structured dissociation tracking developmental lineage, metabolic state, and niche identity, with class-specific diagnostic signatures (Section 6). These alternatives may contribute to observed heterogeneity; the empirical question is the relative contribution of constraint-based, stochastic, and gradient-based variation, which spatial transcriptomics combined with functional readouts is now positioned to address.

Finally, the therapeutic implications developed in this section are proposed as conceptual classes rather than validated strategies. Direct evidence exists for some elements, including restoration of lysosomal capacity in specific models (Choi et al., 2023), metabolic intervention in others (Ulland et al., 2017). However, the integrated strategy of matching intervention class to inferred checkpoint failure remains prospective. We prioritize restoring metabolic and proteostatic capacity where evidence for execution failure is clearest, followed by targeted modulation of tissue-derived gating where specific inhibitory signals are mechanistically identified. We view cell replacement as relevant only where intrinsic constraints cannot be modified pharmacologically (Section 5). The framework is intended to generate testable hypotheses for these interventions, not to claim current readiness for translation.

## 6 Open questions and testable predictions

If microglial heterogeneity reflects constrained transitions rather than interchangeable states, the central problem shifts. The goal is to define how these constraints are established, how they can be measured, and whether they can be experimentally modified. This shifts the focus from identifying additional states to determining the rules that govern state accessibility. A key strength of the checkpoint framework is that it generates falsifiable predictions. If transitions are constraint-limited, then perturbing those constraints should alter functional execution, not just gene expression. Critically, these effects should differ from simple shifts in transcriptional magnitude.

A first question concerns developmental licensing. How is it encoded, and can it be modified in the adult brain? Although ontogeny clearly restricts microglial competence, it remains unclear which aspects of this restriction are fixed and which remain plastic. The checkpoint framework makes a prediction here. Lineage-distinct microglial populations exposed to the same stimulus should differ in transition completion. They should not differ only in marker intensity, but in functional execution. Cells should differ in their ability to sustain outputs such as phagocytosis or resolution. A shift in marker intensity would be consistent with many models. A difference in transition completion would support developmental licensing as a causal constraint. Testing this will require approaches that link developmental lineage to adult function through longitudinal and lineage-resolved analyses (Utz et al., 2020; Barry-Carroll et al., 2023; Brioschi et al., 2023), combined with functional readouts (Fixsen et al., 2023; Saeki et al., 2024; Chen et al., 2026). Concretely, inducible lineage-tracing approaches using *Cx3cr1-CreER* or related systems could be used to compare transition completion across cells with distinct ontogenetic histories, such as resident yolk-sac-derived microglia and monocyte-derived macrophages recruited or repopulating after depletion. The critical readouts would be sustained phagocytic output and resolution kinetics, rather than marker expression alone, as defined operationally in Section 3.1. The AND-logic prediction is that cells with an inappropriate or incomplete developmental history would show impaired transition completion even when acute signaling is engaged.

A second question concerns metabolic and proteostatic capacity. How do these systems define thresholds for transition? The critical prediction is that enhancing lysosomal or autophagic function should rescue execution of a specific functional task. For example, amyloid clearance or inflammatory resolution should improve without altering the upstream transcription. If the signal input is unchanged but the function is restored, capacity is the limiting factor, and this would imply execution rather than signaling. The inverse prediction also holds, meaning that disrupting metabolic systems should block entry into high-demand states while leaving lower-demand homeostatic responses intact. This pattern would indicate selective constraint, and would distinguish a checkpoint from a general stress response (Ulland et al., 2017; Choi et al., 2023; Marino et al., 2023). Experimentally, this can be tested by targeted manipulation of nodes identified in Section 4.2, such as TFEB overexpression or activation to enhance lysosomal capacity, combined with Seahorse-based metabolic flux measurements and quantitative phagocytic assays using defined cargo such as fibrillar amyloid or myelin debris. The key discriminating readout is whether enhancing capacity restores sustained functional output without requiring a proportional increase in upstream receptor-proximal signaling.

A third question concerns how tissue-derived signals are organized to regulate microglial transitions. The hierarchy and integration of niche-derived signals remain poorly defined. The gating framework makes another testable prediction. Modifying specific niche components, such as neuronal inhibition or astrocyte-derived permissive signals, should alter which transitions are accessible (Stogsdill et al., 2022; De Schepper et al., 2023; Yin et al., 2023; Sun et al., 2024). The effect should be selective, and microglia should not be uniformly activated or suppressed. Critically, this predicts strong spatial specificity. Identical perturbations should produce different outcomes in regions. These differences should map onto regional differences in niche composition rather than onto intrinsic microglial heterogeneity alone. Experimental approaches that preserve tissue architecture will be necessary to resolve these relationships. Specifically, organotypic brain slice cultures allow targeted manipulation of niche components while preserving aspects of tissue architecture. Examples include pharmacological modulation of neuronal activity, astrocyte-directed gene manipulation using viral approaches, or enzymatic digestion of specific extracellular matrix components, combined with *in situ* measurements of microglial functional output. The critical prediction is region-specific divergence in outcome: comparable niche perturbations should produce different functional responses depending on local niche composition, with the magnitude or direction of the response tracking spatial heterogeneity in the perturbed component.

A fourth question concerns the reversibility of disease-associated microglial states. If these states reflect incomplete transitions, then relieving the relevant constraint should allow progression. Cells should move toward alternative functional outcomes, including resolution or repair. This can be tested using dynamic measurements of state progression. Perturbations should be combined with longitudinal tracking. Initial empirical progress in this direction has been made using inducible genetic fate-mapping of specific microglial states. Barclay et al. (2024) used a Clec7a-CreERT2 tool to track and manipulate DAM/PAM-like microglial populations in cuprizone-induced demyelination, demonstrating transcriptomic and morphological plasticity of DAM-like cells and a protective role for these cells during remyelination. This work illustrates the type of longitudinal, state-specific manipulation needed to test reversibility, but it also highlights the discriminating test that remains open. Extending this approach, the constraint framework predicts that combining longitudinal fate-mapping with constraint-specific interventions, such as lysosomal capacity enhancement, should reveal whether stalled cells undergo directional transition completion rather than nonspecific bidirectional state drift. The key discriminating prediction is directional. Cells released from a metabolic or proteostatic bottleneck should not simply return to a homeostatic baseline. Instead, cells should complete the transition they had initiated, producing a functional output that matches the target program. Such experiments would distinguish between stable dysfunctional identities and constrained intermediates. The distinction has direct implications for intervention timing.

A fifth question concerns the expansion of the accessible state space. The framework developed here focuses on constraints that limit transitions within an existing developmental repertoire. It remains unclear whether this repertoire can be expanded. Epigenetic reprogramming approaches, forced expression of pioneer transcription factors, or exposure to heterologous developmental signals may enable microglia to access states outside their normal trajectory (Gosselin et al., 2017; Fixsen et al., 2023; Hamagami et al., 2025). This would extend the range of responses rather than restore existing ones. The checkpoint model predicts that such expansion would require modifying the regulatory landscape established during developmental licensing. It cannot be achieved by simply relieving an inhibitory signal. The resulting cells would differ qualitatively, not just quantitatively, from those produced by conventional activation. This connects to emerging work in microglial cell engineering and iPSC-derived microglia, where the question of whether *in vitro*-specified cells recapitulate the full functional repertoire of endogenous microglia remains unresolved (Mancuso et al., 2019; Xu et al., 2020). Testable perturbations include forced expression or restoration of transcription factors such as SALL1 or MAFB, as well as CRISPR-based epigenetic editing at microglia-defining enhancers. The discriminating readout would be whether these interventions expand the accessible response repertoire, enabling functional programs that are weak, absent, or unstable in untreated adult microglia, rather than simply amplifying existing responses.

A sixth question concerns how prior experience shapes microglial constraints (Perry and Holmes, 2014; Wendeln et al., 2018). The checkpoint framework predicts that responses will exhibit path dependence. Identical stimuli should produce different outcomes depending on developmental history, aging, or prior exposure to stress or injury (Hayes et al., 2022; Hata et al., 2023; Kim et al., 2024). These differences should be traceable to specific changes in epigenetic modifications, metabolic remodeling, or altered niche relationships, not variation in the initiating signal. Experimental manipulation of preconditioning variables should alter not only baseline states but also the direction and completeness of subsequent transitions. If confirmed, this would show that prior experience reshapes the constraint landscape. It would also establish that these changes are mechanistically identifiable and potentially reversible. Experimentally, this can be tested through sequential stimulation paradigms, such as low-dose LPS preconditioning followed by amyloid exposure, or by comparing matched injury paradigms across juvenile and adult animals. ATAC-Seq and CUT&RUN for defined histone marks at candidate enhancer regions would provide molecular readouts of changes in the constraint landscape. The discriminating prediction is that responses to the second stimulus, or to the same injury at different developmental stages, should correlate with altered chromatin accessibility or enhancer-associated histone marks, rather than being explained solely by differences in acute signaling thresholds.

Finally, and cutting across all of the above, a central translational challenge is to define how microglial competence can be measured *in vivo*. This question deserves particular emphasis because the entire framework stands or falls on whether constraint, as opposed to signal availability, can be independently assessed. Current approaches rely largely on static transcriptional profiling (Thrupp et al., 2020; Caglayan et al., 2022; Marsh et al., 2022), which cannot distinguish a cell that has not received an activating signal from one that has received it but lacks the capacity to act. Emerging approaches are beginning to close this gap. Spatial transcriptomics combined with functional histological readouts can link transcriptional state to local phagocytic activity, lysosomal load, or inflammatory signaling within preserved tissue architecture (Gazestani et al., 2023; Mallach et al., 2024). *Ex vivo* platforms using human brain slices now allow direct measurement of functional responses in cells whose transcriptional state can be profiled (Adam et al., 2026). These platforms provide a route to testing whether the dissociation between transcriptional activation and functional execution predicted by the constraint framework is observable in human disease tissue. In particular, tool compounds that recapitulate specific microglial states offer a direct test of the fourth prediction above (Tuddenham et al., 2024). If a compound induces a disease-associated transcriptional profile, the constraint framework predicts that functional execution will vary with the donor cell’s prior history, dissociating transcriptional state from functional competence in human tissue. Furthermore, iPSC-derived microglia transplanted into chimeric models offer a complementary approach, enabling controlled comparison of cells with defined developmental histories within shared tissue environments (Mancuso et al., 2019; Xu et al., 2020; Douvaras et al., 2024; Davtyan et al., 2026).

These platforms enable the operationalization of competence defined in Section 3.1. Sustained phagocytic activity under defined stimulus conditions, metabolic flux measurements including oxygen consumption, substrate utilization, and mitochondrial membrane potential, resolution kinetics of inflammatory signaling, and proteomic indicators of completed rather than initiated biosynthetic programs each provide functional outputs that can be compared with transcriptional activation. The checkpoint framework predicts that these outputs will dissociate from transcriptional activation at points of constraint failure.

The diagnostic prediction is class-specific. Cells limited by developmental licensing should show transcriptional activation without the sustained output expected from a fully accessible regulatory architecture. Cells limited by metabolic or proteostatic capacity should initiate but fail to sustain energetically or biosynthetically demanding responses. Cells limited by tissue-derived gating should show stimulus-appropriate transcriptional responses without the spatial or temporal coherence normally imposed by permissive niche signals. These class-specific signatures define the empirical targets that competence measurements must resolve.

The checkpoint framework predicts that such readouts will dissociate from transcriptional profiles in disease-relevant conditions (Figure 1). Cells may appear transcriptionally activated while remaining functionally stalled. This dissociation will carry the most diagnostic and therapeutic information. The convergence of spatial, functional, and human platforms suggests that this prerequisite, while demanding, is now methodologically within reach. Whether the constraint framework holds at the scale of human neurological disease is an empirical question that current technologies are positioned to address.
